# Quantifying individual influence in leading-following behavior of Bechstein’s bats

**DOI:** 10.1038/s41598-020-80946-2

**Published:** 2021-01-29

**Authors:** Pavlin Mavrodiev, Daniela Fleischmann, Gerald Kerth, Frank Schweitzer

**Affiliations:** 1 Chair of Systems Design, ETH Zurich, Weinbergstrasse 56/58, 8092 Zurich, Switzerland; 2 Applied Zoology and Nature Conservation, University of Greifswald, Loitzer Strasse 26, 17489 Greifswald, Germany

**Keywords:** Ecology, Computational models, Animal behaviour

## Abstract

Leading-following behavior as a way of transferring information about the location of resources is wide-spread in many animal societies. It represents active information transfer that allows a given social species to reach collective decisions in the presence of limited information. Although leading-following behavior has received much scientific interest in the form of field studies, there is a need for systematic methods to quantify and study the individual contributions in this information transfer, which would eventually lead us to hypotheses about the individual mechanisms underlying this behaviour. In this paper we propose a general methodology that allows us to (a) infer individual leading-following behaviour from discrete observational data and (b) quantify individual influence based on methods from social network analysis. To demonstrate our methodology, we analyze longitudinal data of the roosting behavior of two different colonies of Bechstein’s bats in different years. Regarding (a) we show how the inference of leading-following events can be calibrated from data making it a general approach when only discrete observations are available. This allows us to address (b) by constructing social networks in which nodes represent individual bats and directed and weighted links—the leading-following events. We then show how social network theory can be used to define and quantify individual influence in a way that reflects the dynamics of the specific social network. We find that individuals can be consistently ranked regarding their influence in the information transfer. Moreover, we identify a small set of individuals that play a central role in leading other bats to roosts. In the case of Bechstein’s bats this finding can direct future studies on the individual-level mechanisms that result in such collective pattern. More generally, we posit that our data-driven methodology can be used to quantify leading-following behavior and individual impact in other animal systems, solely based on discrete observational data.

## Introduction

Leading-following behavior is prominent in different species to transfer information from informed to naïve individuals^1–5^. Those individuals who actively explore their environment, gather private information about the availability or the location of a certain resource, and subsequently lead naïve individuals to these resources^6^. By following a leader, naïve individuals gather information socially and become informed without having to spend prior search effort^7^. When grouping at the resource is beneficial, e.g. during communal roosting, informed individuals benefit from leading naïve individuals as this increases the likelihood of conspecifics being present at the resource^8^.

A natural question in the study of leading-following behaviour is how individuals assume their roles as leaders, followers or both. Studies in collective motion have already reported that distinct leadership roles can emerge if some individuals are more active or better informed than others^4,9^ or stand to gain more from imposing their preferences^10,11^. The presence of a small fraction of informed leaders has also been shown to be sufficient in guiding the movement of large groups with great accuracy in both human and animal societies^12,13^. Some animal studies have even suggested that in addition to immediate cost and benefits, leadership is a personality trait independent of differences in information or knowledge of the environment (see Johnstone and Manica^14^ and references therein). One distinct characteristic of field studies on collective motion, however, is that group movements are tracked continuously^5,15^. Consequently, factors such as proximity, association and communication patterns or dominance, are observed with high enough resolution to inform a reliable picture of how leading-following behavior emerges and what roles different individuals assume.

Continuous tracking, however, is resource intensive and for some social systems prohibitively laborious, such as in the case of fission-fusion systems, in which the group is only temporarily cohesive. Therefore many field experiments collect only discrete observational data in the form of individual recordings of presence at experimental locations. To study leading-following behaviour in such cases, one needs to (a) first reconstruct that behaviour from the available data and (b) only then quantify the resulting leading-following patterns. It is the aim of this paper to propose a sound methodology to accomplish both of these goals.

To demonstrate our methodology, we focus on two colonies of Bechstein’s bats (*Myotis bechsteinii*), a forest-living, European bat species. Using discrete observational data spanning five years, we infer leading-following events by parametrizing empirical insights from field studies and calibrating these parameters from the existing data. In this way, we achieve a statistically robust inference that is entirely data-driven.

After inferring such leading-following events, we then turn to the established field of social network analysis^16^. We represent the information transfer resulting from leading-following conceptually as an information network and then quantify individual impact in it. In the resulting networks individuals are represented as *nodes* and their leading-following events as *directed and weighted links*, where the weights indicate the frequency of such events. This abstraction allows us to further analyze topological characteristics of such networks. On the level of the animal group (here, bat colony), this includes features such as connectedness, i.e. to what extent the information flow resulting from leading-following behavior permeates the whole system. On the individual level, it allows us to calculate centralities to proxy the importance of the nodes, which translates to the influence of specific bats on the information flow.

We note that social network theory has already transcended the human domain and has become widely accepted as an important conceptual framework for studying social interactions in animal groups in general^17–20^. In Bechstein’s bats, social network theory has unveiled the presence of long-term social relationships despite the high fission-fusion dynamics of the colonies, thereby imparting novel insights on the relation between cognitive abilities and social complexity^21^.

Therefore, we see our two-step approach of quantifying individual influence in leading-following as a sound method to study leading-following behavior based on discrete observations. As we also point out in the concluding discussions, we see the potential for a much broader application to studying leading-following behavior in different species, as well.

## Study animals: Bechstein’s bats

### Coordination in roosting behavior

During summer adult female Bechstein’s bats form colonies to communally raise their young (adult males are solitary; Kerth and König^22^). Such maternity colonies comprise 10–50 individuals, have a very stable individual composition, and are highly heterogeneous with respect to the age, reproductive status and the degree of relatedness among colony members^21,23,24^. Colonies switch communal roosts (tree cavities and bat boxes) almost daily and regularly split into several subgroups that use separate day roost^21,22^. Communal roosting provides the females and their offsprings with grouping benefits, such as energetic advantages through clustering (e.g. social thermoregulation^25,26^).

At the same time the frequent roost switching forces the female Bechstein’s bats to regularly explore new potential roosts during their nightly foraging trips and to coordinate their movements among day roosts in order to avoid permanent fission of the colony^27,28^. Experienced individuals, who have discovered the locations of suitable roosts through independent exploration, transfer their private knowledge to naïve conspecifics by leading them to these locations^3^. Such leading-following events take place when one or several experienced bats arrive together with one or several naïve bats at a box at night. Information transfer about suitable roosts provides benefits to both the leading and the following bat. By leading conspecifics to potential roosts, an experienced individual increases the likelihood of communally roosting with conspecifics. At the same time, by following experienced individuals, naïve bats gather information socially without the need to spend prior search effort.

### Field data collection

From 2007 to 2011, we studied two colonies (BS and GB2) of Bechstein’s bats within their natural home ranges located in two forests near Würzburg, Germany (Figure S1, left). The BS colony was not monitored in 2008. Since 1996, all adult female bats in both colonies have been individually marked with individual RFID-tags in their first year of life^29^. Each RFID-tag is programmed with a unique 10-digit ID that can be identified and recorded by automatic reading devices^3^. The study period in each year was between the beginning of May and end of September. In that time, the colonies’ home ranges were equipped with about 20-30 experimental bat boxes per year in addition to a large number of already existing boxes (about 100; Fleischmann et al.^27^; Figure S1, right). These boxes were to serve as day roosts, similar to natural roosts in tree cavities, in which the Bechstein’s bats spend the day. All experimental boxes were equipped with RFID-loggers that recorded the bats’ nightly visits^3,27^. In this way, every time a bat passes the entrance of an experimental box, its unique ID would be read and stored by the reading device without disturbance to the individual.

At the beginning of the study period in each year, the experimental boxes were placed within the home ranges and thus their locations were unknown to the bats until the first colony members discover them through private information gathering. Moreover, each box was positioned within 300 meters of day roosts used in the previous year.

Importantly, not all experimental boxes were discovered by the colony in a given year. Moreover, not all discovered and visited experimental boxes were subsequently used as day roosts.

Our datasets, thus, consist of the yearly recordings of the reading devices from all experimental boxes for each of the two colonies in each of the 5 years. Each recording contains a timestamp and the unique 10-digit ID of the bat who activated the reading device. An example dataset is shown in Table S1 in the supplemental material. Table 1 shows a summary of the total number of readings and the number of installed, discovered and occupied experimental roosts, for each colony throughout the years.

**Table 1:** Data summary.

Colony	Year	colony size	#readings	#boxes installed	#boxes discovered	#boxes occupied
GB2	2007	31	1002	17	11	4
	2008	34	4243	32	32	25
	2009	21	1273	21	16	6
	2010	44	878	17	12	3
	2011	16	1929	18	18	6
BS	2007	16	5600	25	20	12
	2009	17	9102	32	28	16
	2010	19	2169	23	19	7
	2011	7	2016	20	13	9

A box is defined as discovered, if at least one naïve individual is recorded in it during the season. When at least 2 bats (minimum number for a group decision) used a new box as a day roost, this box was considered occupied by a group of bats.

## Methodology

### Inferring leading-following networks

#### Defining leading-following events

Unlike studies on collective motion where group movement is tracked continuously^5,15^, our datasets contain only discrete records of bat appearances at experimental boxes. Quantifying individual influence is, thus, contingent on a rigorous method for inferring leading-following events from discrete recordings of animal occurrences. To denote the information that individuals possess about the location of experimental boxes, we refine the nomenclature used by Kerth and Reckardt^3^. An individual bat is said to be naïve at time ${{{\mathbf {t}}}}_{{{\mathbf {1}}}}$ regarding a given box, if it has *not* been recorded by the reading device in that box for all times ${{\mathbf {t}}}<{{{\mathbf {t}}}}_{{{\mathbf {1}}}}$. Similarly, an individual bat is considered experienced at time ${{{\mathbf {t}}}}_{{{\mathbf {2}}}}$ regarding a given box, if it has been recorded in that box at any previous time ${{\mathbf {t}}}<{{{\mathbf {t}}}}_{{{\mathbf {2}}}}$. We define a *leading-following (L/F) event* to a given box at time ${{{\mathbf {t}}}}_{{{\mathbf {3}}}}$ as the joint visit of two individuals—one naïve and one experienced at time ${{{\mathbf {t}}}}_{{{\mathbf {3}}}}$. The details of how joint arrivals are calculated are presented later in the paper.

In case more than two bats arrive jointly, we form all possible L/F pairs consisting of one naïve follower and one experienced leader. In case the leader and the follower were recorded multiple times, we take those times that minimize the difference between their appearances in the dataset (see Table S2 and associated explanation). Finally, we refer to *time*_*difference* of an L/F event as the absolute difference between the recording times of the leader and the follower.

With this definition of L/F events, the actual inference of L/F event patterns from the data relies on three parameters: (1) *lf_delay*: the maximum allowed time difference (in minutes) between consecutive recordings of a leader and a follower, (2) *turnaround_time*: the minimum time (in minutes) an experienced bat in an L/F event needs to potentially become a leader, i.e. the time needed to find and lead followers, and (3) *occupation_deadline*: the hour in the morning on the day of a box occupation, after which subsequent recordings from this box are ignored because of swarming behavior (example of local enhancement, Kerth et al.^3^, Kerth and Reckardt^28^).

#### Calibrating parameter values

It is important to note that each of the three parameters affects the inference of L/F events differently. Values of *lf_delay* that are too large would lead us to incorrectly define many visits of an experienced and naïve bats as joint visits, i.e. as legitimate L/F events, even those that occur in different days. Too small values of *turnaround_time*, on the other hand, will force us to “break” one L/F event with one leader and multiple followers into separate L/F events where the previous followers are now falsely deemed as leaders. Similarly, if *occupation_deadline* is too late in the morning, a lot of the joint visits due to swarming will be incorrectly inferred as L/F events. We discuss the parameter influence on the inference procedure in more detail in Section S.3.

To choose appropriate values for the three parameters we resort to a purely data-driven process based on comparing the distributions of L/F event time differences statistically. This represents a more rigorous approach compared to an otherwise subjective calibration based on observations often used in analysis of field studies.

**Figure 1:**
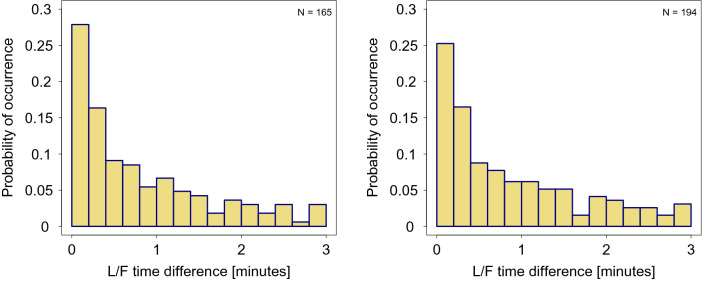
L/F time differences for the GB2 colony in 2008. Histograms show the absolute differences between the times at which the leader and the follower were recorded in all identified L/F events. Parameters: turnaround_time = lf_delay = 3 min (both plots),occupation_deadline = 2 a.m. (left) and occupation_deadline = 3 a.m. (right). Insets indicates the total number of identified L/F events.

Empirical research in the field of information transfer in Bechstein’s bats has suggested 3 min for *lf_delay* and 3 a.m. for *occupation_deadline* as a reasonable rule of thumb (Kerth and Reckardt 2003). We build upon these heuristics by (a) introducing an additional parameter *turnaround_time* defined above and (b) by comparing the distributions of time differences of all L/F events (Fig. 1).

Note that any combination of the three parameters is a 3-tuple, which generates a set of L/F time differences from all identified L/F events in the dataset. In Fig. 1 we show two-dimensional histograms of L/F time differences for fixed values of *lf_delay* = *turnaround_time* = 3 min, and *occupation_deadline* = 2 a.m. (left) and *occupation_deadline* = 3 a.m. (right). As there is no objective method to quantify the behaviour underlying each of the parameters, we argue that L/F time differences best capture the effect that varying the parameters has on the L/F events we identify. For example, a visual inspection of Fig. 1 hints that increasing *occupation_deadline* from 2 a.m. to 3 a.m. does not change the time difference distributions. This implies that swarming has not yet set in (otherwise, we would expect quantitatively more events with longer time difference), and the additional L/F events on the right-hand side are genuine. Consequently, we would prefer *occupation_deadline* = 3 a.m., as it increases our sample size. Section S.4 details the expected effects of swarming on the distributions of L/F time differences.

The core of our method revolves around pairwise testing for statistical difference in the distributions of L/F time differences generated by different values of the 3-parameter tuple. We start from the reasonable default values mentioned above and summarize the result of the calibration in Tables 2 and 3.

**Table 2:** GB 2 colony in 2008 with lf_delay = 5 min.

Turnaround_time	Occupation_deadline pairs					
	2 a.m./3 a.m.	2 a.m./5 a.m.	2 a.m./8 a.m.	3 a.m./5 a.m.	3 a.m./8 a.m.	5 a.m./8 a.m.
2	0.725/0.362	0.522/0.261	**0**.**005**/**0**.**003**	0.782/0.391	**0**.**011**/**0**.**006**	**0**.**012**/**0**.**006**
3	0.619/0.31	0.349/0.175	**0**.**006**/**0**.**003**	0.671/0.335	**0**.**019**/**0**.**009**	**0**.**03**/**0**.**015**
5	0.457/0.229	0.135/0.068	**0**/**0**	0.47/0.235	**0**.**004**/**0**.**002**	**0**.**018**/**0**.**009**
7	0.457/0.228	0.094/**0**.**047**	**0**/**0**	0.36/0.18	**0**.**002**/**0**.**001**	**0**.**015**/**0**.**008**
9	0.514/0.257	0.085/**0**.**043**	**0**/**0**	0.29/0.145	**0**.**001**/**0**	**0**.**012**/**0**.**006**

Wilcox rank-sum test was performed with 10^3^ bootstraps. Table cells are formatted as *p*_1_/*p*_2_, where *p*_1_ and *p*_2_ are the p-values for the hypotheses $\mathcal{H}_{1}$ and $\mathcal{H}_{2}$, respectively. Bold values indicate that the corresponding null hypotheses (see main text) can be rejected in favor of the alternatives ones at a statistically significant level.

To generate sufficient sample sizes for the comparison, the dataset we chose to analyze was the GB2 colony in 2008 (Table 1). The reason is that, in 2008, the colony had the highest number of discovered and occupied boxes, the second largest colony size, and a large amount of individual readings. Therefore, we expected to identify the largest number of L/F events from this dataset, and thus obtain the most robust parameter values.

In Table 2, lf_delay is fixed at 5 min, while occupation_deadline is varied in {2 a.m., 3 a.m., 5 a.m., 8 a.m.}, and turnaround_time – in {2, 3, 5, 7, 9} min. For each value of turnaround_time (rows in the table), we compare the time difference distributions (${\mathcal {X}}_{i}$/${\mathcal {Y}}_{i}$) between all possible pairs of occupation_deadline. The comparison is done via a bootstrapped Wilcoxon rank-sum test on the null hypothesis that the two distributions are the same, against the two-sided alternative ${\mathcal {H}}_{1}$, and the one-sided alternative ${\mathcal {H}}_{2}$ that ${\mathcal {X}}_{i} < {\mathcal {Y}}_{i}$. Each table cell shows the *p* value for the two-sided and one-sided test, respectively.

As an example, fixing $\texttt {turnaround\_time}=2$ min, we see that the distribution of L/F time differences for occupation_deadline at 2 a.m. is not statistically different from the distribution with occupation_deadline at 3 a.m. (*p* value = 0.725). This is an indication that the nature of the identified L/F events is invariant to the later deadline, hence it is unlikely that we have inadvertently included swarming effects. Further inspection of the table reveals that qualitative changes in L/F time differences occur when occupation_deadline = 8 a.m., but not for the other pair-wise comparisons. The one-sided test indicates the type of these changes, namely that L/F events inferred up to 8 a.m. on the day of occupation, tend to have larger time differences compared to earlier occupation deadlines. This is in line with the reasoning in Section S.4 of the Supplementary Material and implies the presence of swarming effects. Therefore, occupation_deadline = 8 a.m. is likely too late.

Moreover, this conclusion holds when varying turnaround_time, as well. The impact of this parameter on the L/F time differences seems to be small, in the range considered. The effect of turnaround_time is primarily on the number of identified L/F events, as assuming larger recruitment delays excludes events where the leader found a follower relatively quickly (Table 3).

**Table 3:** Number of identified L/F events for the GB2 colony in 2008 with different values of the three parameters.

Occupation_deadline	lf_delay = 3					lf_delay = 5				
	Turnaround_time					Turnaround_time				
	2	3	5	7	9	2	3	5	7	9
2 a.m.	173	165	158	155	154	211	201	185	181	181
3 a.m.	202	194	184	181	178	245	235	221	217	206
5 a.m.	274	269	249	248	234	329	321	298	297	290
8 a.m.	354	349	326	325	321	456	440	411	410	405

Considering these arguments, we see that lf_delay = 5 min, turnaround_time = 3 min. and occupation_deadline = 5 a.m. provide the best trade-off between maximising the number of identified L/F events while still keeping the distribution of L/F time difference undistorted by swarming. We also see these values as improvements over the common heuristics mentioned in the beginning of the section.

#### Constructing leading-following networks

With the parameters calibrated following the above procedure, we identified all L/F events in each of our datasets (that is 5 datasets for colony GB2 and 4 datasets for colony BS2 for all years, Table 1).

We then constructed directed and weighted leading-following (L/F) networks from each dataset. In these networks, a node represents an individual bat and a link between two nodes indicates their involvement in a leading-following event. More specifically, links are *directed*. A directed link from node A to node B, denoted as A $\rightarrow$ B, means that individual A followed individual B to a given experimental box. The weight of this directed link is the number of times that A followed B (to different experimental boxes) during the study period in the respective year. Note that in constructing these L/F networks, we ignore the target box of each L/F event and simply sum up the number of L/F events to compute the link weights.

We also compute the number of weakly connected (WCC) and strongly connected components (SCC). A WCC of a network is a sub-network in which any node can be reached from any other node, either by a link between these two nodes, or by following a sequence of links through other nodes, regardless of the direction of these links. Similarly, a SCC is a WCC with the additional restriction that the *direction of the links* must be respected when connecting any two nodes. As we explain in the next section, these two measures are particularly important for judging the extent to which information can spread in a network.

### Social network analysis

#### Quantifying individual influence

**Figure 2:**
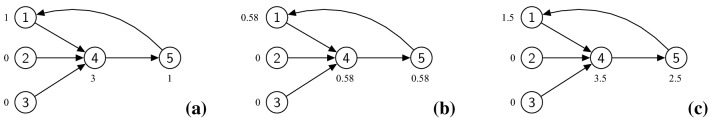
Differences between the three candidate centrality measures. The centralities for each measure are indicated next to each node. (**a**) *In-degree centrality*. Here, only direct influence is measured. Individual 4 is most influential, as she spread information to three different individuals. Individuals 1, and 5 with one follower each, have still equal importance. (**b**) *Eigenvector centrality*. Since individuals 2 and 3 have no followers, they are attributed zero influence, and thus contribute nothing to the influence of their leader, individual 4. In turn, 1, 4, and 5, each have one follower of non-zero importance, hence they have the same eigenvector scores. (**c**) *Second-degree centrality* with $\alpha=0.5.$ Individual 4 has a higher centrality than her in-degree score, as we account for the indirect contribution of individual 1 $(3 + 0.5 \times 1 = 3.5).$ However, 5 is now more important than 1, because 4 contributes to 5 indirectly $(1+0.5 \times 3 = 2.5).$

We can now use the *topology* of the constructed networks, i.e. the relation between nodes expressed by their links, to characterize the position of individuals in such a network. Our aim is to identify those nodes, i.e. individual bats, that are most influential in leading other bats. In social network analysis, the importance, or influence, of a node in a certain dynamical process flowing through the network is referred to as *centrality*. There are various centrality measures in use, and each makes certain implicit assumptions about the dynamical process flowing through the network^30^. Choosing a centrality measure is, thus, context-dependent (see Fig. 2). An improperly selected centrality metric, can lead to losing the ability to interpret the measure correctly, this way deducing wrong answers.

#### In-degree, eigenvector and second-degree centrality

In our case, an appropriate centrality measure must reflect the notion of individual influence in spreading information about suitable roosts. If influence is best proxied by the total amount of roosts that a given bat made known to the colony, then a suitable centrality measure is the *in-degree* centrality (Fig. 2a). This quantity measures individual importance as the total number of bats that an experienced bat spreads information to directly, i.e. the number of L/F events in which an individual participated as a leader. In-degree centrality is, thus, calculated as the weighted sum of all directed links that point to a given experienced individual.

In-degree centrality measures the total number of leadings, i.e. direct influence, *without* considering how the information distributed by a leader to its followers propagates further through the colony. To also account for such indirect effects, an alternative centrality measure is *eigenvector centrality* (Fig. 2b). In a social network, a node has high eigenvector centrality if it is pointed to by nodes that themselves have high eigenvector centralities. In other words, an experienced bat leading a few bats, who themselves lead a lot can be more influential than a bat leading many other bats who in turn never lead. The computation of eigenvector centralities is presented in Section S.5 of the Supplementary Material.

The in-degree and eigenvector centralities represent two extremes, the former measuring exclusively direct influence, and the latter additionally measuring *all* possible indirect ways, in which information can flow from one individual to all the rest. Eigenvector centrality, however, considers information chains of all lengths to be of equal importance, *regardless* of the target experimental box. An information chain of length *k* is simply a sequence of *k* L/F events identified for a given network, in which the follower in a previous L/F event is the leader of a later L/F event.

For example, two identified L/F events A $\rightarrow$ B and C $\rightarrow$ A, constitute a chain of length two (in addition to forming two separate chains of length one). Assuming both L/F events were to the same experimental box, then B ought to obtain direct importance from having led A, but also indirect contribution, for were it not to B, A would not have learned about this box and thus could not lead C to it. This assumption is not entirely correct, however, since it is possible, though unknowable, that A would have found the roost by its own exploration, or that A “forgot” the information obtained from B, and re-visited the box before leading C. The latter issue is exacerbated with the length of the event chains we consider. However, if the two L/F events were not to the same target box, B should not obtain any indirect benefits from the second L/F event. Note that in-degree centrality only considers L/F chains of lenth 1, i.e. direct influence.

Since we construct aggregated L/F networks (i.e. links represent leading-following, *disregarding* the target box), we risk attributing too much importance to individuals when using eigenvector centrality. The metric will simply grow with the length of the chain and individuals who are part of longer chains will tend to be quantified as more influential. This would risk distorting individuals’ influence scores, since it is highly unlikely that a long L/F chain had the same target box for all L/F events in the chain.

**Figure 3:**
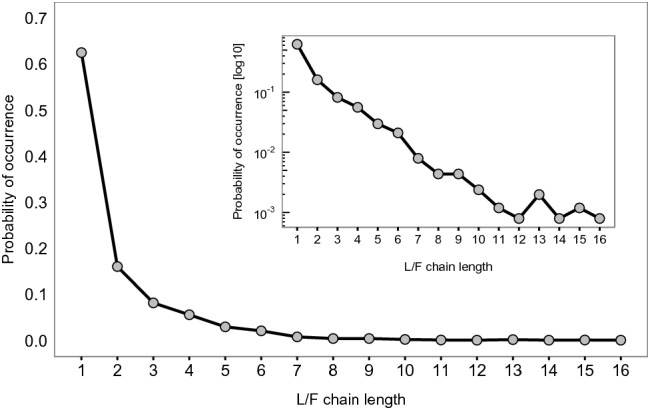
Probability distribution of the lengths of L/F event chains, calculated over all nine datasets. Inset: log-linear plot of the data.

We again use our data to inform the proper balance between direct and indirect influence, and thus to choose the right centrality metric. Figure 3 shows the relative frequency, aggregated over all datasets, of observing chains of L/F events. This frequency can be interpreted as the probability of finding chains of a given length. As the inset in Fig. 3 demonstrates, the probability distribution resembles an exponential distribution. The plot further indicates that chains longer than 16 did not occur in any of the datasets we have. More importantly, event chains of length up to two constitute more than 80% of all lengths observed, and the probability of longer chains decreases drastically. We, thus, posit that limiting the influence computation to L/F chains of maximum length of two reflects properly the majority pattern observed in the data. Hence, it represents a proper heuristic that minimizes the risk of inflating indirect influence scores by including long L/F chains that do not represent genuine information spread about the same roost.

For this reason, we define a new metric—*second-degree centrality* (Fig. 2c)—which computes centrality as the in-degree of the focal individual and the sum of the in-degrees of its followers, weighted by a factor $\alpha$ (in that sense the followers of one’s followers are its second-degree followers). This reflects our observation that chains of length up to two constitute the majority in all datasets. We, thus, use second-degree centrality with $\alpha =0.5$ as the main measure for quantifying individual influence. We will, however, keep in-degree and eigenvector centrality for comparison purposes to make sure that our heuristic metric does indeed reflect a balance between the two extrema and produces consistent results.

All analysis was done in the R programming language.

### Ethics approval

Handling and tagging of the bats were conducted under the permits for species protection (55.1-8642.01-2/00) and animal welfare (54-2531.01-56/06; 55.2-2531.01-79/10; 55.2-2531.01-47/11) that had been issued by the government of Lower Franconia.

## Results

### Constructing the L/F networks

As already explained, an L/F network illustrates all detected L/F events for a given colony in a given year, where nodes represent individual bats and directed links represent leading-following events. The data is aggregated over the whole season, thus the width of the links indicates the number of events in the dataset.

We construct the networks for *all datasets* and present a summary of their salient network characteristics in Table 4, regarding their degree of connectedness. Network density is defined as the fraction of inferred L/F events out of the maximum possible number of L/F events for that network. For example, the L/F network for the GB2 colony in 2007 consists of 31 individuals, hence the maximum possible number of L/F events is $31\times 30=930$, which yields a network density of 0.06.

**Table 4:** Topological characteristics of the leading-following networks from the GB2 and BS colonies.

Colony	Year	#bats	#L/F events	density	#WCC	#SCC	size of largest SCC
GB2	2007	31	60	0.07	4	23	9
	**2008**	**34**	**262**	**0.23**	**1**	**2**	**33**
	2009	21	33	0.08	2	19	3
	2010	44	142	0.08	1	22	14
	**2011**	**16**	**86**	**0.35**	**1**	**2**	**15»**
**BS**	**2007**	**16**	**169**	**0.70**	**1**	**1**	**16**
	**2009**	**17**	**201**	**0.74**	**1**	**1**	**17**
	**2010**	**19**	**148**	**0.43**	**1**	**3**	**17**
	**2011**	**7**	**26**	**0.62**	**1**	**1**	**7**

Shown are number of bats (nodes), number of identified L/F events (links), network density, number of weakly connected components, number of strongly connected components, and the size of the largest strongly connected component. Rows in bold are the dataset we consider for further analysis.

We see that the two colonies differ in this respect through the years. While the L/F networks for the BS colony display high density and connectivity for all study years, the L/F networks for GB2 colony in the years 2007, 2009 and 2010 have low density consistent with the fewer L/F events observed. Therefore, to calculate the importance of each individual, we use only the cyan-coloured datasets in Table 4, as they provide the most reliable sample sizes of detected L/F events for statistical analysis.

If we focus only on these datasets, we also find that the information spreading through the colony was prominent enough to give us enough data for quantitative conclusions. We see that their respective L/F networks are weakly connected, that is there is only *one* weakly connected component, which means that *all* individuals participated in L/F events, i.e. in information spreading. Moreover, these networks consist of only a few (1-3) strongly connected components (SCC). Within an SCC, each individual can be reached from any other individual by following (a chain of) *directed* links. In most of the chosen cases, the size of the largest SCC is similar to the total number of nodes, which means that the vast majority of individuals participated as *both* leaders and followers. Otherwise, one could reach a given individual through a directed chain, but will not be able to connect from this individual back to the network via a directed chain. Hence, individuals would be part of a weakly connected component (WCC) because they are *either* followers *or* leaders, but they would not be part of a SCC.

### Quantifying individual influence

**Figure 4:**
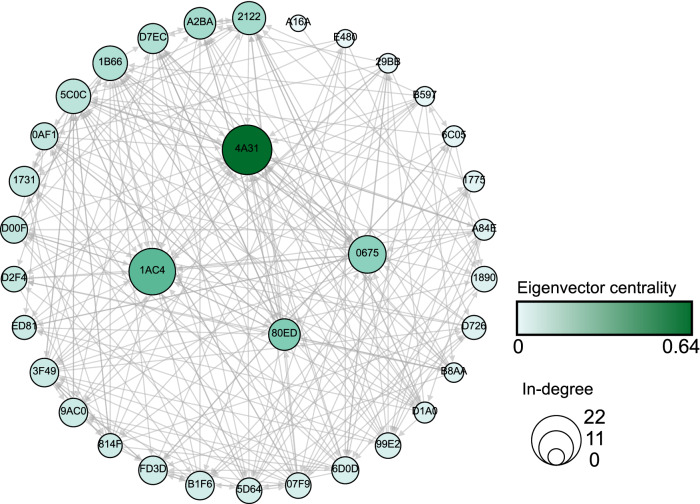
Aggregated leading-following network for the GB2 colony in 2008. Nodes represent individual bats (indicated by a hexadecimal number inside the circle). Directed links represent following behaviour. Node colors indicate eigenvector centrality, whereas node sizes indicate in-degree centrality. The four individuals with highest eigenvector centrality are shown in the middle. Note that for the sake of illustration, links show only *unique* L/F events. I.e. leading-following between the same leader and follower, but to different roosts, are omitted to maintain the readability of the graph. Total number of unique L/F events is 262, while the total number of L/F events, including multiple leading-following between the same individuals, is 321 (Table 4).

As an illustration, Fig. 4 shows one L/F network for the GB2 colony in the year 2008. It is clear that individuals differ remarkably with respect to their importance, as reflected both by their in-degree centrality (size of the nodes) and their eigenvector centrality (node color). It is also evident that there are correlation between in-degree and eigenvector centrality, as visible for the four individuals in the center.

We now use the constructed networks to quantify the importance of individuals for spreading information about suitable roosts. To this end, we compute the three different centrality measures introduced in “Social Network Analysis” section—in-degree centrality, eigenvector centrality and our reference measure, second-degree centrality.

**Figure 5:**
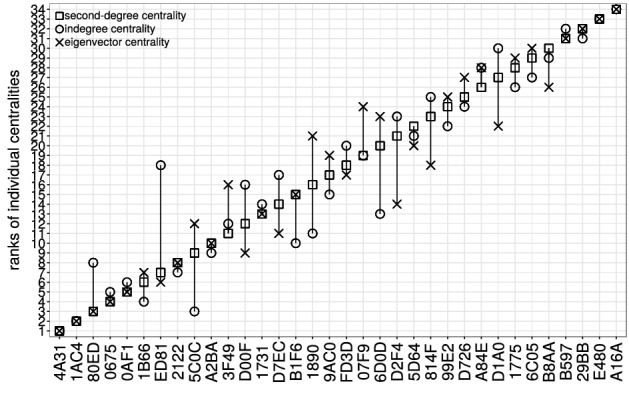
Ranked individual influence of bats of colony GB2 in year 2008. The x-axis displays the last four digits of a bat’s unique identification number. The y-axis displays the rank according to the second degree centrality (square symbols) in increasing rank order (rank 1—highest centrality). For each bat we additionally plot its rank when importance is quantified as in-degree (circle symbols) and eigenvector centrality (cross symbols). Overlap of the three symbols indicates that the given individual has the same rank, regardless of the centrality measure used. For the individual centrality values see Figure S2.

**Figure 6:**
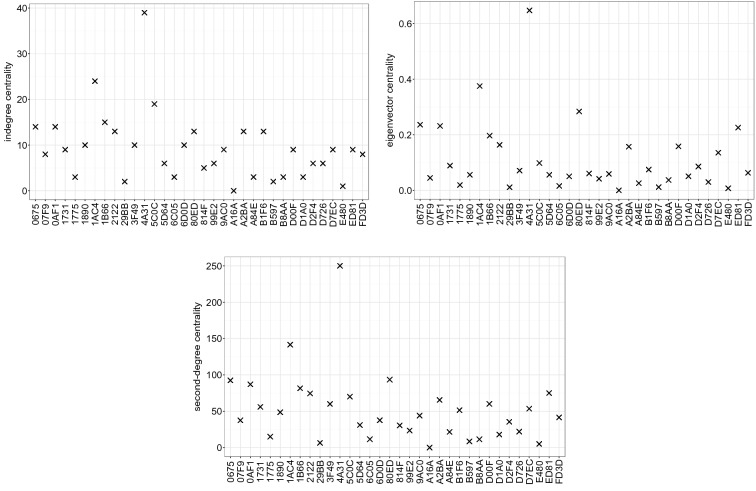
Individual influence quantified according to the three centrality measures introduced in “Social Network Analysis” section: (top left) in-degree centrality, (top right) eigenvector centrality, (bottom) second-degree centrality (top left). For the calculation, the L/F network constructed from the dataset of colony GB2 in year 2008 was used. The x-axis displays the last four digits of a bat’s unique identification number.

Figure 5 shows the results of each of these measures separately for the colony GB2 for the year 2008. If we compare the *absolute values* of the centralities, we find that influence scores are heterogeneous with a *majority* of individuals exerting low to mid influence and a *minority* having high influence. Importantly, this result holds for all considered datasets. We can use the absolute values to determine the *relative* importance, by ranking individuals according to their second-degree centrality. The results are shown in Fig. 6, where the diagonal indicates increasing rank, i.e. decreasing importance. In order to determine whether these results are robust if instead of second-degree centrality the other two measures are used for the ranking, we have provided the respective ranks in the same plot. As Fig. 6 shows, the three proposed centrality measures produce a highly consistent ranking of individual influence.

To verify this finding, we have extended the above analysis to all datasets indicated in Table 4. For each dataset, we have then calculated the Pearson correlation between the rankings obtained from the three centrality measures. The results are given in Table S.7 in the Supplementary Material. We find that for all datasets the Pearson correlation is very high for all combinations. That means that when considering aggregated measures, such as rankings, second-degree centrality produces consistent rankings. However, as we have argued, it differs from in-degree and eigenvector centrality on the individual level, as it more accurately captures the extent to which the information about a given roost travels along L/F event chains observed in the data.

## Discussion

In this paper we have provided a systematic data-driven method to quantify individual contributions for active information transfer in the form of leading-following behavior. We demonstrated our approach on datasets of discrete recordings from two colonies Bechstein’s bats.

Individual contributions in information transfer can tell us a lot about the complexity of social organizations in different animal systems. For example, in African elephants, a single matriach leads a group and the group members profit from following her as she has long-term experience^31^. In primates, the individual influence of group members can depend on the context, and may range from a single dominant individual who influences where a group moves to, to a more widely distributed influence on travel destinations among group member^32,33^.

We have addressed two main challenges present when working with datasets of discrete observations. First, we presented a robust method to infer genuine leading-following events from raw recordings of individual presence and second we applied social network theory as a conceptual framework to represent the information flow from leading-following and to ultimately quantify individual influence.

Regarding the first challenge, we note that most field experiments, including ours, are limited by the state-of-the-art passive RFID-tagging, which only records presence data. There is a more advanced technique^15^ that uses a proximity sensor system to continuously track the leading-following behaviour between female bats and their juvenile to suitable roosts. However, such technology is still in its nascent stage and not widely used in field experiments, as with this battery-powered system small bat species cannot be tagged at present and it is not possible to follow many individuals over an extended period of time.

Our methodological contribution can be also adopted for other species where leading-following behavior plays a role and only recordings of individual positions are available. This includes, for example, automatic RFID-tag recordings at feeding stations^34^ and other resources where different group members meet, such as burrows in rodents^35^. As we demonstrate, such recordings can be systematically analyzed by comparing (statistically) the distributions of L/F time differences, to infer genuine L/F events.

A major contribution in addressing the first challenge is a thorough investigation of the parameters that allow to distinguish a L/F event from other types of encounters (e.g. local enhancement) at a given box. We recall that there is no ground truth available that informs us about the correct identification of L/F events from the data. We argued that the time differences of L/F events can be used to calibrate three relevant parameters: (1) the maximum allowed time difference (in minutes) between consecutive recordings of a leader and a follower, (2) the minimum time (in minutes) an experienced bat in an L/F event needs to potentially become a leader, i.e. the time needed to find and lead followers, and (3) the hour in the morning on the day of a box occupation, after which subsequent recordings from this box are ignored because occupation is considered to have already taken place.

Regarding the second challenge, we have proposed a new measure of individual influence that can be derived from the L/F networks we construct—*second-degree* centrality. Importantly, proxying influence as a centrality score goes beyond the widely used association indexes and the corresponding Mantel tests in studying animal systems. Association indexes are *local* measures in that they only reflect dyadic relations between any two individuals. To quantify the *systemic* influence of individuals, we need to provide measures that also capture their proclivity to act as social hubs, as recognized already by Farine and Whitehead^34^ and Brent^36^. In this respect, we see the application of social networks for analyzing information spreading as an excellent choice.

Our method differs also from common techniques^34^ to study animal association patterns via social networks. Typically, when social networks are used, the *observed interaction strength* between two individuals is either thresholded (i.e. it exists only if interaction strength is above threshold), sampled or used as a link weight, to calculate various association indexes^17,37–40^. In line with Farine and Whitehead^34^ (see specifically Fig. 2 therein), we do *not* threshold our networks to avoid dubious statistical biases. Instead, we include *all* of the observed individuals and their recorded activity and analyze the *full scale* of inferred interactions.

Note that we focus our analysis on dense networks (see Table 4). We found that these networks have only one weakly connected component (WCC) which contains most of the individuals. Density is a proxy for the intensity of leading-following behaviour, while the presence of one large WCC indicates that the majority of the colony partook in leading-following. Moreover, we also found that in most cases there are only very few (1-3) strongly connected components (SCC) of different size in the network (see Table 4).

Hence, we can conclude that individuals in the same SCC participated both as leaders and as followers in different events. This tells us that information about suitable roosts is not concentrated in only a few important individuals, but is spread across the whole colony.

At the same time, we could also detect that not all individuals play an *equal* role as leaders or followers. Instead, their influence, measured by leading inexperienced bats, differs considerably. To quantify these differences, we used different centrality measures as proxies of importance. Two of these, in-degree and eigenvector centrality, are established measures, while the third one, second-degree centrality is a new measure introduced by us. As explained in “Social Network Analysis” section, it provides a proper balance between direct and indirect influence in the information transfer.

Computing the different centralities for each individual, we could identify that there are only a few important individuals that lead most of the other bats. These individuals stand out regardless of the centrality measure used. In particular, we also calculated that there are significant correlations between the rankings obtained by using the different centrality measures. We emphasize that measuring influence by means of centralities cannot be simply reduced to comparing numbers of leading events. The latter would not allow us to distinguish whether individuals always lead the same or diverse followers, or whether such followers are of less or equal importance in comparison to the leader.

We believe that our results can guide future empirical and theoretical studies in two ways. First of all, we should realize that the constructed L/F networks do not already tell us about the *mechanisms* by which pairs of leaders and followers are formed. This process, known as *recruitment*, can be revealed by testing different recruitment rules in computer simulations, to check whether they result in the importance scores obtained from the empirical networks. In essence this entails the development of various *null models*. Null models are recognized as useful tools to test the viability of these recruitment rules in the presence of inherently non-independent behavioral data^41^. We investigate a variety of such null models about recruitment behavior in Bechstein’s bats in^42^.

Secondly, additional field work needs to be devoted to study the behavioural variability of individuals in playing their role as leaders or followers. For example, demographic, health or genetic characteristics can influence such roles^43–46^. With our study, however, we have already identified those individual bats that are prominent in these roles. This allows to target future experiments particularly toward individuals with very high or very low influence, to find out how different characteristics impact their leading-following behavior.

## Supplementary Information

Supplementary Information.
