# Supplementary material for: Quantifying individual influence in leading-following behavior of Bechstein’s bats
Source: Sci Rep. 2021 Jan 29;11:2691. doi: 10.1038/s41598-020-80946-2 (PMC7846810; doi:10.1038/s41598-020-80946-2)
Supplement: Supplementary file 1 — Supplementary Information. [file 41598_2020_80946_MOESM1_ESM.pdf]

Quantifying individual influence in leading-following behavior of Bechstein's bats

Pavlin Mavrodiev[1], Daniela Fleischmann[2], Gerald Kerth[2], Frank Schweitzer[1] *

[1]Chair of Systems Design, ETH Zurich,
Weinbergstrasse 56/58, CH-8092 Zurich, Switzerland
[2] Applied Zoology and Nature Conservation, University of Greifswald,
Loitzer Strasse 26, 17489 Greifswald, Germany

Electronic Supplementary Information

S.1 Home ranges (left) and experimental bat boxes (right)

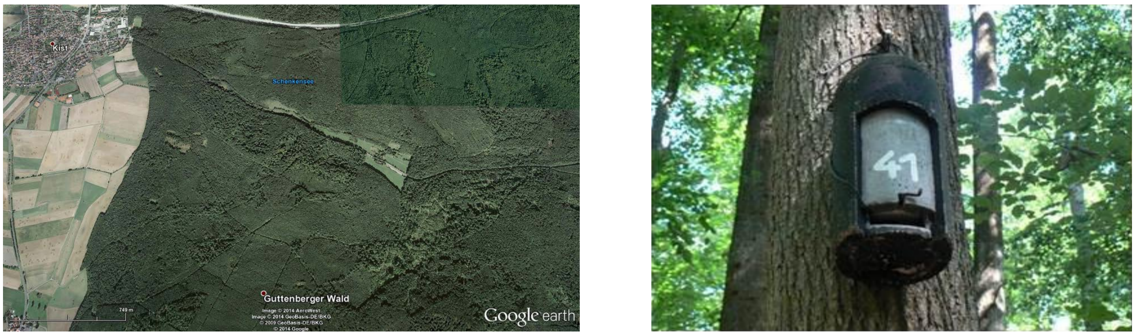

Figure **S1**: **Left**: Home ranges. **Right**: Experimental box

S.2 Illustration of the raw recordings in our datasets

S.3 Inferring L/F events

This Section outlines in more details the effects of the three parameters introduce in Section 3.1 of the main text on the inference of L/F events.

The first parameter **lf_delay** represents the maximum time difference allowed between consecutive recordings of a leader and a follower, regardless of the order. It is important in determining

* Corresponding author, `fschweitzer@ethz.ch`

Table S1: An excerpt from the recordings of an experimental box for the GB2 colony in year 2008. Line numbers serve as a visual guide only and are not part of the data. Each line corresponds to one reading, i.e. one activation of the reading device by a visiting bat. Columns are separated by semicolon. The first column shows the date of the reading (in this case June 2nd), the second column indicates the time of the recording in 24-hour format, the third column contains the unique 10-digit ID of each bat, and the last columns is a status message.

```
Ser.-Nr.: [0401]
02.06;00:50:25;00065db1f6;OK
02.06;01:00:47;00068e1ac4;OK
02.06;01:00:51;00068e1ac4;OK
02.06;01:00:52;00068e1ac4;OK
```

which patterns constitute a joint visit of two individuals, as bats do not enter a box immediately upon arriving: females returning at night to a day roost usually encircle it several times before entering (Kerth and Reckardt, 2003; SchÃűner *et al.*, 2010). Therefore, **lf_delay** limits the sheer number of L/F events we detect, since the higher the limit, the more likely it is to find an experienced and a naïve individual recorded within **lf_delay** of each other. In the limit of **lf_delay** $\rightarrow \infty$, we would detect the maximum number of L/F events, many of which would be false positives, as bats recorded days apart would still be assumed to have "jointly" arrived at a box.

The second parameter **turnaround_time** represents the *minimum* time a follower in an L/F event needs to potentially become a leader, i.e. the time needed to find, recruit, and lead other followers. The importance of this parameter becomes apparent in Table S2, which shows a frequently occurring recording pattern.

Table S2: A simplified example of how turnaround_time affects the inference of L/F events

```
02.06;01:00:00;00065db1f6;OK
02.06;01:00:20;00068e1ac4;OK
02.06;01:01:00;00068e1ac4;OK
02.06;01:01:01;00065ded81;OK
```

Assume that, for this box, individual **00065db1f6** is experienced at time **01:00:00** (line 1), individual **00068e1ac4** is naïve at **01:00:20** (line 2), and individual **00065ded81** is naïve at **01:01:10** (line 4). Taking **lf_delay**=3 minutes (which is a good rule-of-thumb (Kerth and Reckardt, 2003) we can deduce that individual **00068e1ac4** followed individual **00065db1f6** to that box, i.e. **00068e1ac4**$\rightarrow$**00065db1f6**. More precisely, we infer an L/F event to this box

with the leader recorded at **01:00:00** and the follower at **01:00:20**. The time difference of this event is 20 seconds.

Let us further assume that **00068e1ac4** liked the box she was just led to, and in turn would like to show it to other individuals. Its second recording in this dataset is on the third line - 40 seconds after its first appearance as a follower. If we assume that **turnaround_time** < 40 seconds, then we also have to assume that **00068e1ac4** would have had enough time to fly within its home range, meet other individuals, recruit and ultimately lead them back to this box. In this example, she led individual **00065ded81** who appeared within a time of **lf_delay** from it, i.e. we then also have to infer the L/F event **00065ded81→00068e1ac4**. In addition, however, we see that **00065db1f6** and **00065ded81** appear within **lf_delay** of each other, hence we must also form the L/F pair **00065ded81→00065db1f6**. Evidently this last L/F event contradicts **00065ded81→00068e1ac4**. Hence, **turnaround_time** < 40 seconds is a wrong assumption.

The issue is that, in reality, the 40-second delay between the two readings of **00068e1ac4** is most likely not due to it having led another individual to the box. Instead, it is highly likely that either (i) the first reading showed the bat entering the box and then leaving it again shortly thereafter or (ii) that the bat was simply encircling the box for 40 seconds, and then triggered the reading device a second time upon re-entry. The proper distinction between actual recruitment and such behavioural variability is the role of the parameter **turnaround_time**. In the toy example from Table S2 a more realistic interpretation is that **00065db1f6** led both **00068e1ac4** and **00065ded81**, i.e. we would only infer two L/F events. Note that since **00068e1ac4** appears twice, we associate the time of its first recording (**01:00:20**) with the L/F event **00068e1ac4→00065db1f6**, since it minimizes the time difference to the recording of the leader.

The third parameter **occupation_deadline** is the hour in the morning, on the day of a box occupation, after which subsequent recordings from this box are *ignored*. The necessity to ignore some recordings comes from the need to distinguish between genuine information exchange about suitable roosts (in terms of leading-following) and "pre-occupation" behaviour. Before the occupation of a given box, experienced individuals who have decided to roost there, fly around the box and emit echolocation calls that attract naïve individuals to the same box (O'Shea and Vaughan, 1977; SchÃűner *et al.*, 2010). It has been suggested that this broadcasted information is used by naïve bats (especially juveniles) to learn the location of suitable roosts from experienced conspecifics (Kerth *et al.*, 2003). The result is that occupation is preceded by a growing group of individuals (experienced and naïve) flying around, or *swarming*, the roost for several hours. In our data, this is reflected by readings of naïve individuals, which appear shortly after each other in a long sequence, together with the readings of experienced bats. As a result, additional L/F events will be identified with time differences close to the allowable limit of **lf_delay** (see Section S.4 for illustration). These L/F events do not constitute genuine recruitment, in the sense

that naïve individuals were led to a roost, but rather reflect the swarming phenomenon (local enhancement). Therefore, we need a temporal deadline on the day of a box occupation, after which subsequent readings in this box are attributed to swarming, and thus ignored.

S.4 Effects of swarming on L/F time differences

Below we illustrate how swarming affects L/F time differences. In particular, the time differences, in presence of swarming, are skewed towards the **lf_delay** limit. Table S3 shows a typical recording pattern from a representative experimental box close to 6am on the day of the box occupation. Here, five experienced and five naïve individuals were recorded within about 10 minutes. Table **S4** contains all L/F events identified from the sample with **lf_delay** fixed at 5 minutes.

Table S3: An excerpt from the *processed* recordings of an experimental box for the GB2 colony in 2008. Each line contains the individual information status, recording time, and unique identification number, in that order, separated by semi-colon.

```
1.  NAIVE      ;05:52:28;00068E1731
2.  EXPERIENCED;05:53:52;00060F6D0D
3.  NAIVE      ;05:54:41;00064407F9
4.  EXPERIENCED;05:55:02;0005FDFD3D
5.  EXPERIENCED;05:56:12;00065EA84E
6.  NAIVE      ;05:57:22;00060D6C05
7.  EXPERIENCED;05:59:03;0006979AC0
8.  NAIVE      ;06:00:31;0006011890
9.  EXPERIENCED;06:02:22;00064407F9
10. NAIVE      ;06:02:32;0006011890
```

How do we identify L/F events from such data, without confounding them with swarming behavior? In fact, we are not interested in identifying swarming behavior. Instead, we only want to extract legitimate L/F events. In Section **??**, we already analyzed the distributions of time differences between L/F events, to detect for which parameter values the distributions start to deviate significantly. This is sufficient to calibrate the three parameters of our method.

Here we also offer a possible explanation for these deviations. In Table S3, the mean time difference of the events is 2.4 minutes, and the minimum is strictly above 1 minute. We argue that this characteristic is more consistent with swarming behaviour, in which a few experienced individuals attract naïve conspecifics by circling around the roost and emitting echolocation calls. Since experienced and naïve individuals are not grouped together as in genuine leading-following pairs, it takes time for a naïve individual to respond to the calls and fly to the roost. As a result, most L/F events identified in this way tend to have larger time differences closer to the allowable limit

of **lf_delay**. We use precisely this observation when fine-tuning the **occupation_deadline** parameter.

Table **S4**: The L/F events corresponding to the recording pattern in Table S3. Parameters `lf_delay`=5 minutes and `occupation_deadline`=8am. `turnaround_time` does not affect this example, as no naïve individual appears again as a leader.

Follower	Leader	L/F time difference	L/F times
00060F6D0D	00068E1731	1.4	05:53:52 / 05:52:28
00064407F9	00068E1731	2.21667	05:54:41 / 05:52:28
0005FDFD3D	00068E1731	2.56667	05:55:02 / 05:52:28
00065EA84E	00068E1731	3.73333	05:56:12 / 05:52:28
00060F6D0D	00060D6C05	3.5	05:53:52 / 05:57:22
00064407F9	00060D6C05	2.68333	05:54:41 / 05:57:22
0005FDFD3D	00060D6C05	2.33333	05:55:02 / 05:57:22
00065EA84E	00060D6C05	1.16667	05:56:12 / 05:57:22
00065EA84E	0006011890	4.31667	05:56:12 / 06:00:31
0006979AC0	00060D6C05	1.68333	05:59:03 / 05:57:22
0006979AC0	0006011890	1.46667	05:59:03 / 06:00:31
00064407F9	0006011890	1.85	06:02:22 / 06:00:31

As a comparison, consider the recording pattern in Table S6 from another box close to 2am on the day of its occupation. The L/F events corresponding to this pattern are shown in Table **S5**. The mean time difference is 1.5 minutes and the minimum is zero, as individual arrivals exceeded the time resolution of the reading device. This indicates that an experienced individual did appear close together with a designated follower. As for the couple of events with large time differences, they are most likely due to naïve individuals remaining at the entrance of the box, thereby triggering the reading device repetitively, than to swarming. As seen from Table S6, three such individuals, **00065DB1F6**, **000697D00F** and **00068E1B66**, generate long recording sequences that prevent other followers from examining the box upon arrival, thereby forming an L/F event with large time difference to the leader.

Table **S5**: The L/F events corresponding to Table S6. Parameters as in Table **S4**.

Follower	Leader	L/F time difference	L/F times
0005FE0AF1	000697D00F	0	01:58:54 / 01:58:54
00068E1B66	000697D00F	0	01:58:54 / 01:58:54
0005FE0AF1	00064380ED	3.45	01:58:48 / 01:55:21
00068E1B66	00064380ED	3.55	01:58:54 / 01:55:21
000697A2BA	00064380ED	4.68333	02:00:02 / 01:55:21
0005FE0AF1	00065DB1F6	0.216667	01:58:56 / 01:59:09
00068E1B66	00065DB1F6	0.15	01:59:00 / 01:59:09
000697A2BA	00065DB1F6	0.866667	02:00:02 / 01:59:10
000697A2BA	000697D00F	1.13333	02:00:02 / 01:58:54

Table S6: An excerpt from the *processed* recordings of an experimental box for the GB2 colony in 2008. The pattern is formatted as in Table S3.

```
1.  NAIVE      ;01:55:21;00064380ED
2.  NAIVE      ;01:55:25;00065DB1F6
3.  NAIVE      ;01:55:26;00065DB1F6
4.  NAIVE      ;01:55:36;000697D00F
5.  NAIVE      ;01:55:49;00065DB1F6
6.  NAIVE      ;01:55:50;00065DB1F6
7.  NAIVE      ;01:55:51;00065DB1F6
8.  NAIVE      ;01:55:52;00065DB1F6
9.  NAIVE      ;01:55:55;00065DB1F6
10. NAIVE      ;01:57:12;00065DB1F6
11. NAIVE      ;01:57:14;00065DB1F6
12. NAIVE      ;01:57:21;000697D00F
13. NAIVE      ;01:57:22;000697D00F
14. NAIVE      ;01:57:23;000697D00F
15. NAIVE      ;01:57:25;000697D00F
16. NAIVE      ;01:57:27;000697D00F
17. NAIVE      ;01:58:30;000697D00F
18. NAIVE      ;01:58:31;000697D00F
19. NAIVE      ;01:58:32;000697D00F
20. NAIVE      ;01:58:35;000697D00F
21. NAIVE      ;01:58:36;000697D00F
22. NAIVE      ;01:58:45;000697D00F
23. NAIVE      ;01:58:46;000697D00F
24. NAIVE      ;01:58:47;000697D00F
25. EXPERIENCED;01:58:48;0005FE0AF1
26. EXPERIENCED;01:58:49;0005FE0AF1
27. EXPERIENCED;01:58:50;0005FE0AF1
28. EXPERIENCED;01:58:52;0005FE0AF1
29. NAIVE      ;01:58:53;000697D00F
30. EXPERIENCED;01:58:54;0005FE0AF1
31. EXPERIENCED;01:58:54;00068E1B66
32. NAIVE      ;01:58:54;000697D00F
33. EXPERIENCED;01:58:55;0005FE0AF1
34. EXPERIENCED;01:58:56;0005FE0AF1
35. EXPERIENCED;01:58:56;00068E1B66
36. EXPERIENCED;01:58:57;00068E1B66
37. EXPERIENCED;01:58:58;00068E1B66
38. EXPERIENCED;01:58:59;00068E1B66
39. EXPERIENCED;01:59:00;00068E1B66
40. NAIVE      ;01:59:09;00065DB1F6
41. NAIVE      ;01:59:10;00065DB1F6
42. EXPERIENCED;02:00:02;000697A2BA
```

S.5 Eigenvector centrality explained

Consider the following simple leading-following network with 4 bats (nodes) and 5 leading-following events (links):

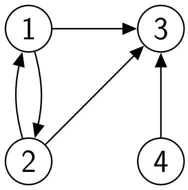

An alternative representation of this network is through its so called *adjacency* matrix, A, which indicates which two nodes are adjacent to each other.

$$A = \begin{pmatrix} 0 & 1 & 0 & 0 \\ 1 & 0 & 0 & 0 \\ 1 & 1 & 0 & 1 \\ 0 & 0 & 0 & 0 \end{pmatrix}$$

The elements $a_{i,j}$ (i, and j index rows and columns, respectively) in this matrix are 1 if a directed link exists between nodes j and i. In other words, $a_{i,j} = 1$ if j followed i. Otherwise, $a_{i,j} = 0$. For example the first column $a_{i,1}$ gives all nodes that node 1 follows. We see that $a_{2,1} = a_{3,1} = 1$, so 1 has followed both 2 and 3.

The main idea behind eigenvector centrality is that the centrality of a node i, c_i, is proportionate to the sum of the centralities of all nodes who follow it. Staying with node 1, its centrality is the sum of the centralities of nodes 2 and 3, i.e. $c_1 = \frac{1}{\lambda}c_2 + \frac{1}{\lambda}c_3$ or $\lambda.c_1 = c_2 + c_3$ for some proportionality constant λ. In this way, we can express the centralities of all nodes and write them as a system of equations:

$$\begin{array}{rcrcrcrcr} \lambda \cdot c_1 & = & 0 \cdot c_1 & + & 1 \cdot c_2 & + & 1 \cdot c_3 & + & 0 \cdot c_4 \\ \lambda \cdot c_2 & = & 1 \cdot c_1 & + & 0 \cdot c_2 & + & 0 \cdot c_3 & + & 0 \cdot c_4 \\ \lambda \cdot c_3 & = & 1 \cdot c_1 & + & 1 \cdot c_2 & + & 0 \cdot c_3 & + & 1 \cdot c_4 \\ \lambda \cdot c_4 & = & 0 \cdot c_1 & + & 0 \cdot c_2 & + & 0 \cdot c_3 & + & 0 \cdot c_4 \end{array}$$

In matrix form the above system can be rewritten as:

$$\lambda \cdot \begin{pmatrix} c_1 \\ c_2 \\ c_3 \\ c_4 \end{pmatrix} = \begin{pmatrix} 0 & 1 & 0 & 0 \\ 1 & 0 & 0 & 0 \\ 1 & 1 & 0 & 1 \\ 0 & 0 & 0 & 0 \end{pmatrix} \cdot \begin{pmatrix} c_1 \\ c_2 \\ c_3 \\ c_4 \end{pmatrix}$$

or in vector notation: $\lambda \cdot \vec{c} = A \cdot \vec{c}$. This is the familiar eigenvector problem. We need to find a vector $\vec{c}$ such that upon applying matrix A to it, the result is a scaled version of $\vec{c}$ with a scaling

factor λ. The unknown vector $\vec{c}$ is called an eigenvector of the matrix A, and λ is referred to as the eigenvalue, which corresponds to that eigenvector. Solving the system of equations yields: $\vec{c} = \{0.408, 0.408, 0.816, 0\}$ and $\lambda = 1$. Therefore node 3 is most central since it is followed by everyone. Nodes 1 and 2 follow each other so they boost their own centrality, and node 4 is not followed by anyone so its centrality is 0.

S.6 Correlations between centrality measures

Table **S7**: Correlations between the rankings produced by the three different centrality measures. S stands ranking using second degree centrality, E stands for ranking using eigenvector centrality, and D stands for ranking using in-degree centrality. The first column lists the different datasets as described in Table 1 of the main text.

	$S \Leftrightarrow E$	$S \Leftrightarrow D$	$D \Leftrightarrow E$
GB2 2008	0.98	0.97	0.90
GB2 2011	0.99	0.93	0.92
BS 2007	0.99	0.98	0.98
BS 2009	0.99	0.97	0.98
BS 2010	0.99	0.96	0.94
BS 2011	0.99	0.93	0.94
